# Determinants of joint stiffness and jumping height during drop jump

**DOI:** 10.14814/phy2.15678

**Published:** 2023-06-06

**Authors:** Takehiro Kosaka, Shuhei Sasajima, Ayaka Yasuda, Soushi Mino, Keitaro Kubo

**Affiliations:** ^1^ Department of Life Science The University of Tokyo, Meguro Tokyo Japan

**Keywords:** active muscle stiffness, medial gastrocnemius muscles, tendon stiffness, ultrasonography

## Abstract

The present study aimed to examine the effects of muscle‐tendon mechanical properties and electromyographic activity on joint stiffness and jumping height and to explore the determinants of joint stiffness and jumping height. Twenty‐nine males performed unilateral drop jumps at three drop heights (10, 20, and 30 cm) using only the ankle joint on the sledge apparatus. Ankle joint stiffness, jumping height, and electromyographic activity of the plantar flexor muscles were measured during drop jumps. Active muscle stiffness of the medial gastrocnemius muscle was calculated according to changes in the estimated muscle force and fascicle length during fast stretching at five different angular velocities (100, 200, 300, 500, and 600 deg s^−1^) after submaximal isometric contractions. Tendon stiffness and elastic energy were measured during ramp and ballistic contractions. Active muscle stiffness was significantly correlated with joint stiffness, except for a few conditions. Tendon stiffness measured during ramp and ballistic contractions was not significantly correlated with joint stiffness. The ratios of electromyographic activity before landing and during the eccentric phase to that during the concentric phase were significantly correlated with joint stiffness. In addition, jumping heights at 10 and 20 cm (except for 30 cm) drop heights were strongly associated with the tendon elastic energy, whereas no other measured variables showed significant correlations with jumping heights. These results suggested that (1) joint stiffness is determined by active muscle stiffness and electromyographic activity patterns during jumping, and (2) jumping height is determined by tendon elastic energy.

## Graphical abstract

o date, there is no unanimous view on the determinants of joint stiffness and jumping height. The present results suggest that joint stiffness is determined by active muscle stiffness and electromyographic activity patterns, while jumping height is determined by tendon elastic energy. These findings will contribute to the establishment of training methods to improve stretch‐shortening cycle performance.

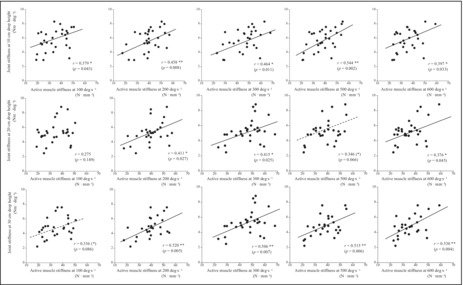

## 1 INTRODUCTION

Joint stiffness is known to play an important role in performance and efficiency during stretch‐shortening cycle exercises (Arampatzis et al., 2001; Chelly & Denis, 2001; Farley & Morgenroth, 1999). Previous studies identified neuromuscular activity and muscle‐tendon mechanical properties as factors that define joint stiffness (e.g., Horita et al., 1996). Regarding neuromuscular activity, several studies showed that electromyographic activities before landing and during the eccentric phase of drop jumps were strongly associated with joint stiffness (Hoffren et al., 2007; Horita et al., 2002). Furthermore, it has been shown that joint stiffness reduction after repetitive stretch‐shortening cycle exercises is due to decreased electromyographic activity and stretch reflexes (Horita et al., 1996; Kuitunen et al., 2007).

Regarding tendon properties among the muscle‐tendon mechanical properties, we previously reported that joint stiffness was unrelated to tendon stiffness (Kubo et al., 2007). However, this study involved measuring tendon stiffness during ramp contractions with a slow strain rate of the tendon. Recent studies demonstrated that tendon properties measured during ballistic contractions with a high strain rate (similar to that during jumping and running) were markedly different from those during ramp contractions (e.g., Kouno et al., 2021). Therefore, we must investigate the relationship between joint stiffness and tendon properties measured during ballistic contractions.

Since joint stiffness and tendon stiffness were not correlated in our previous study, cited in the last paragraph (Kubo et al., 2007), we expected that muscle mechanical properties would be relevant to joint stiffness. The mechanical properties of human muscles in vivo were evaluated under passive conditions in most previous studies, which further examined the relationship between performance during stretch‐shortening cycle exercise and training‐induced changes in passive muscle stiffness (Ando et al., 2021; Miyamoto et al., 2019; Takahashi et al., 2018). However, to explore the relationship between joint stiffness and muscle mechanical properties during exercise, it is necessary to examine muscle stiffness under active rather than passive conditions. We devised a method to quantify the stiffness of the medial gastrocnemius muscle under active conditions, i.e., active muscle stiffness (Kubo, 2014), and conducted various intervention studies (Kubo et al., 2017, 2021; Kubo & Ikebukuro, 2019; Suzuki et al., 2020). Our recent studies demonstrated that joint stiffness and active muscle stiffness transiently decreased after repeated hopping exercises and increased with 12 weeks of plyometric training, although tendon stiffness did not change with either intervention (Kubo et al., 2017, 2021; Kubo & Ikebukuro, 2019). Therefore, it is expected that there is a relationship between joint stiffness and active muscle stiffness, but the actual relationship between the two has yet to be confirmed. Furthermore, we reported that active muscle stiffness was affected by angular velocity during measurement (Kubo et al., 2020). Therefore, it is likely that joint stiffness during drop jumps at various drop heights may be more related to active muscle stiffness measured under conditions similar to angular velocity during drop jumps.

In general, jumping height is commonly employed as a performance measure of stretch‐shortening cycle exercise (e.g., Bojsen‐Moller et al., 2005). However, it is unclear whether joint stiffness is related to jumping height, although joint stiffness (and leg stiffness) is related to running speed and efficiency (Arampatzis et al., 1999; Heise & Martin, 1998; Kuitunen et al., 2002). Furthermore, in several studies, muscle strength and tendon stiffness have been described as determinants of jumping height (Bojsen‐Moller et al., 2005; Fischer et al., 2017; Pentidis et al., 2020), but no consensus has been reached. One reason for this discrepancy is that most studies employed vertical jumps with compound joint movements, and thus could not identify the primary muscles. Recently, we fabricated a device that can measure the performance of jumping exercises using only the ankle joint. We examined the relationship between single‐joint exercise performance and muscle‐tendon mechanical properties (e.g., Kubo et al., 2021). Using this method, we could overcome the problems described above and identify the determinants of jumping height. Furthermore, jumping height corresponds to the integral of the reaction force in the push‐off phase during jumping. The integral value of the reaction force during this phase is considered to correspond to the amount of energy exerted by the muscle‐tendon complex. Hence, jumping height may be more strongly related to tendon elastic energy than muscle strength and tendon stiffness.

The purpose of this study was to investigate the effects of muscle‐tendon mechanical properties and electromyographic activity on joint stiffness and jumping height, and to explore the determinants of joint stiffness and jumping height during drop jumps. We hypothesized that joint stiffness would be strongly associated with active muscle stiffness (especially under near angular velocity conditions during jumping) and electromyographic activity, while jumping height would be associated with tendon elastic energy.

## 2 METHODS

### 2.1 Participants

The sample size was estimated using data from our preliminary study (*n* = 12) in which the relationships between active muscle stiffness (at 100, 200, 300, 500, and 600 deg s^−1^) and joint stiffness at a 20‐cm drop height were determined. On the basis of an *α* level of 0.05 and a power (1 − *ß*) of 0.8, G*Power (G*Power 3.1.9.3) showed that at least 25 participants were necessary for this study. Twenty‐nine physically active men (age: 23.3 ± 2.0 years, height: 173.5 ± 5.6 cm, body mass: 67.3 ± 9.8 kg, mean ± SD) participated in this study. They were not involved in any type of resistance exercise program for at least 1 year before testing. They were fully informed of the procedures to be utilized, as well as the purposes of the study. Written informed consent was obtained from all participants. This study was approved by the Ethics Committee for Human Experiments, Department of Life Science (Sports Sciences), The University of Tokyo.

### 2.2 Active muscle stiffness

A specially designed dynamometer (T.K.K.S‐18035, Takei Scientific Instruments Co., Ltd.) was used to measure active muscle stiffness using the procedure described in our previous study (Kubo et al., 2020). Participants lay prone on the dynamometer bench, and their torso was secured with a special belt. The ankle joint was set at 100 deg (with the foot perpendicular to the tibia = 90 deg with angles more than 90 deg on plantar flexion), and the knee joint was in full extension. The foot was secured tightly to the footplate of a dynamometer using two straps. After a standardized warm‐up and submaximal contractions, subjects were asked to perform twice 3‐s maximal voluntary contraction (MVC) for plantar flexion. The highest MVC value was used to determine the target torque during the measurement of active muscle stiffness (see below).

After a 5‐min rest period, participants performed active muscle stiffness measurements at five different angular velocities (100, 200, 300, 500, and 600 deg s^−1^). The dynamometer was programmed to apply 100 to 80 deg of dorsiflexion. The measurement of active muscle stiffness was performed three times per each angular velocity at 50% MVC with the visual aid of exerted torque with an oscilloscope. During fast dorsiflexion, participants were asked to maintain the same level of effort (under consciousness) during dorsiflexion rotation. The order of tasks (100, 200, 300, 500, and 600 deg s^−1^) was randomized to avoid any systematic effects. Periods of 140, 80, 60, 48, and 48 ms after the onset of stretch were analyzed at 100, 200, 300, 500, and 600 deg s^−1^ to equalize the analyzed range of motion among the five angular velocities (Kubo et al., 2020). In addition, each angular velocity measurement was performed twice at 0% MVC (relaxed condition). The averaged torque in the relaxed condition was subtracted from the measured torque during the active condition (Allum & Mauritz, 1984; Blanpied & Smidt, 1992; Kubo, 2014). The measured values were the average of three tests. The ankle joint torque measured by the dynamometer (TQ) was converted to muscle force (Fm) by the following equation (e.g., Kubo et al., 2017):

$$\mathrm{Fm}=k\cdot \mathrm{TQ}\cdot {\mathrm{MA}}^{-1}$$

where *k* represented the relative contribution of the physiological cross‐sectional area of the medial gastrocnemius muscle (MG) within the plantar flexor muscles (Fukunaga et al., 1996) and MA was obtained using the tendon excursion method during passive ankle rotation (see below; Tendon mechanical properties), as described by Fath et al. (2010).

During the measurement of active muscle stiffness, the fascicle length of MG was measured using real‐time ultrasonic apparatus (Prosound *α*7, Hitachi Aloka Medical). Ultrasonic images were stored at 100 Hz at 100, 200, and 300 deg s^−1^ and 125 Hz at 500 and 600 deg s^−1^ in the computer memory of the apparatus (Kubo et al., 2020). An electric signal was superimposed on the ultrasonic images to synchronize them with the torque and joint angle. The slope of muscle force–fascicle length was defined as active muscle stiffness (e.g., Kubo, 2014). In our previous study (Kubo et al., 2020), the repeatability of measurement of active muscle stiffness was investigated on 2 separate days involving eight young men. The intraclass correlation coefficient and coefficient of variation were 0.891 and 10.4% at 100 deg s^−1^, 0.829 and 13.5% at 200 deg s^−1^, 0.880 and 11.2% at 300 deg s^−1^, 0.795 and 12.9% at 500 deg s^−1^, and 0.863 and 10.7% at 600 deg s^−1^, respectively. In the present study, the coefficient of variation of three measurements (inter‐day reliability) was 15.7% at 100 deg s^−1^, 12.4% at 200 deg s^−1^, 12.0% at 300 deg s^−1^, 9.4% at 500 deg s^−1^, and 12.2% at 600 deg s^−1^.

### 2.3 Tendon mechanical properties

Tendon mechanical properties (stiffness and elastic energy) at two different strain rates (see below) were measured using the procedure described in our previous studies (e.g., Kubo et al., 2017). Participants lay prone on the test bench with each foot securely fastened to the footplate of a dynamometer (custom‐made, VINE) using two straps. The ankle joint was set at 90 deg, and the knee joint was fully extended. For ramp contraction, participants were asked to gradually increase torque from relaxation to MVC within 5 s. For ballistic contraction, participants were asked to exert isometric torque from relaxation to MVC vigorously and rapidly. The measurements for ramp and ballistic contractions were performed twice with a 1‐min rest between tests.

During the measurement of tendon mechanical properties, ultrasonic images of MG were recorded on a videotape at 60 Hz. The ultrasonic image, torque, and ankle angle (by an electrical goniometer) were synchronized using a timer. Displacement of the intersection point between the fascicle and aponeurosis indicated the lengthening of the tendon. However, the movement of this point was also caused by angular joint rotation occurring in the direction of ankle plantarflexion during “isometric” contraction. To measure ankle joint rotation during the exercises, an electrical goniometer (Penny and Giles) was placed on the lateral aspect of the ankle. Additional measurements were conducted under passive conditions from 90 to 99 deg of the ankle joint to correct the measurements taken for tendon elongation. The movement of this point under passive conditions was subtracted from the measured tendon elongation during isometric contractions (e.g., Magnusson et al., 2001). Only values corrected for angular rotation are presented in this study. In addition, the moment arm length of each participant was obtained using the tendon excursion method (Fath et al., 2010) under passive conditions. The slope of the relationship between tendon excursion (movement of intersection point between the fascicle and aponeurosis) and angular displacement of 9 deg (from 90 to 99 deg) was defined as the moment arm length.

The torque measured by the dynamometer was converted to muscle force using the same method as that used to measure active muscle stiffness. In the present study, the slope of muscle force and elongation of tendon structures above 50% MVC was defined as tendon stiffness (e.g., Kubo et al., 2017). The tendon elastic energy was obtained by calculating the area below the Fm–tendon elongation curve (Kubo et al., 2021). To eliminate the effects of body mass, the maximal muscle force and elastic energy were converted to values relative to body mass. In our previous study (Kubo & Ikebukuro, 2019), the repeatability of measurement of tendon stiffness was investigated on 2 separate days involving eleven young men. The intraclass correlation coefficient and coefficient of variation were 0.924 and 8.2% for ramp contraction and 0.909 and 11.1% for ballistic contraction, respectively. In the present study, the inter‐day reliabilities of the measured variables (two times) were assessed for all participants. Regarding tendon stiffness, the coefficient of variation was 14.4% during ramp and 9.0% during ballistic contractions. Regarding tendon elastic energy, the coefficient of variation was 8.7% during ramp and 9.4% during ballistic contractions.

### 2.4 Joint stiffness and jumping height during drop jump

**FIGURE 1:**
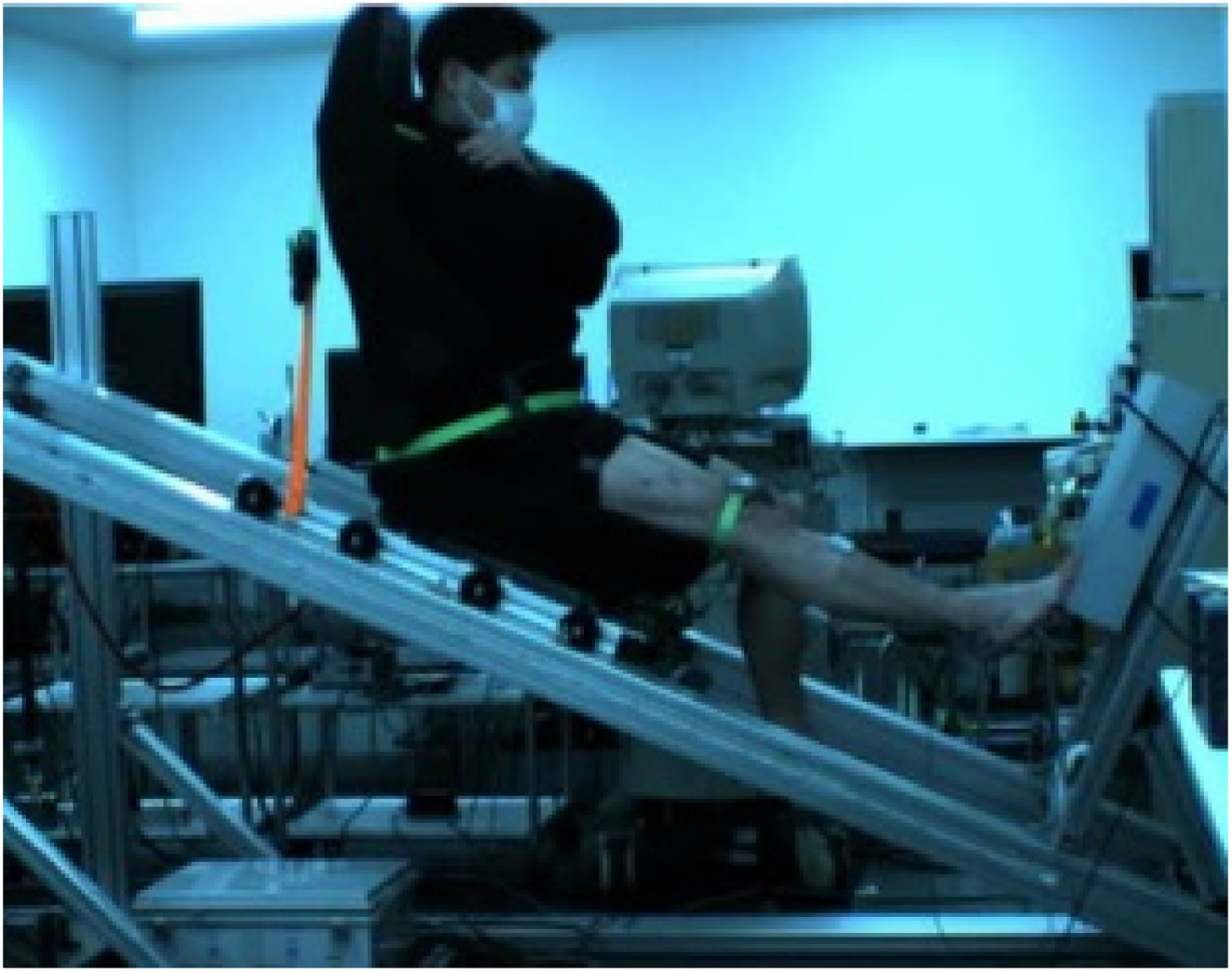
The experimental setup of the measurement of joint stiffness during drop jump.

Participants performed a unilateral drop jump using only the ankle joint on the custom‐designed sledge apparatus (AO‐3000K, Applied Office). The experimental setup is shown in Figure 1. The vertical reaction force during the drop jump was recorded from the force plate (Kistler, 9281B) attached to the footplate of the apparatus. Retroreflective marks were attached to the trochanter major, center of rotation of the knee, lateral malleolus, and fifth metatarsophalangeal joint. During jumping, participants were filmed using a digital high‐speed camera (VCC‐H1600C, Digimo) at a sampling frequency of 250 Hz.

**FIGURE 2:**
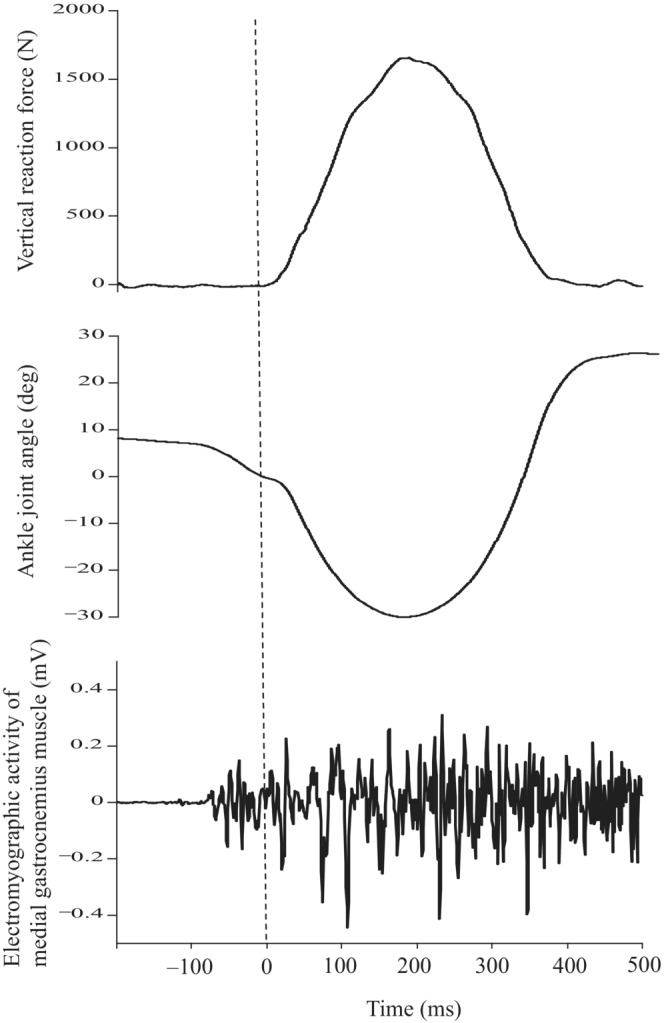
Typical example of the vertical reaction force, ankle joint angle, and electromyographic activity of the medial gastrocnemius muscle during drop jump. The vertical dotted line represents a landing.

Before testing, participants had adequate practice (submaximal jumps) to become accustomed to the test procedure. They were dropped from three drop heights (10, 20, and 30 cm) from the surface of the force plate with assistance using equipment of the sledge apparatus. After landing on the force plate, they stopped the fall motion by exerting eccentrically plantar flexion torque and then immediately started plantar flexion to take off. The test was repeated five times per condition (drop heights of 10, 20, and 30 cm) with at least 1 min between trials. A typical example of the raw data (vertical reaction force, ankle angle, and electromyographic activity) is presented in Figure 2. The jump height and ankle joint angle were measured by motion analysis software (Frame‐DIAS ver. 5, DKH). The data of the ankle angle were filtered with a Butterworth‐type low‐pass filter of the fourth order with a cutoff frequency of 15 Hz. Jump height was assessed as the maximum displacement of the seat of the sledge apparatus from the position at a 90‐deg ankle angle. We excluded trials with slight knee joint flexion (>10 deg) based on images taken by the high‐speed video camera. The averaged values of three trials, excluding the highest and lowest jump heights, were adopted. The range of motion of the ankle from touchdown to the lowest position (ROM) was calculated. The ankle joint torque during the drop jump was estimated from the following equation (Kawakami et al., 2002; Kubo et al., 2007, 2017, 2021):

$$\text{Ankle joint torque}=\mathrm{Fz}\cdot \text{L1}\cdot \cos\;\left(A_{J}\right)$$

where Fz, L1, and A_J_ are the vertical component of the ground reaction force, the length from the estimated center of the ankle joint to ball of the foot, and ankle joint angle, respectively. Ankle joint stiffness was calculated as the change in joint torque divided by the change in the ankle joint angle during the eccentric phase (e.g., Kubo et al., 2007). Unfortunately, two participants failed to properly execute a drop jump with a 30‐cm drop height. In our previous study (Kubo & Ikebukuro, 2019), the repeatability of measurement of joint stiffness was investigated on 2 separate days involving eleven young men. The intraclass correlation coefficient and coefficient of variation were 0.874 and 10.5%, respectively. In the present study, the inter‐day reliabilities of the measured variables (three times) were assessed for all subjects. Regarding joint stiffness, the coefficient of variation was 17.5% at a 10‐cm drop height, 14.3% at a 20‐cm drop height, and 15.2% at a 30‐cm drop height. Regarding jumping height, the coefficient of variation was 5.3% at a 10‐cm drop height, 5.0% at a 20‐cm drop height, and 7.9% at a 30‐cm drop height.

In addition, electromyographic activity (EMG) during jumping was sampled at 1 kHz using a wireless EMG telemeter system (BioLog DL‐5500, S&ME). Surface electrodes (DL‐510, S&ME) were placed on the skin over the muscle belly of MG, lateral gastrocnemius muscle (LG), and soleus muscle (SOL). The raw data were band‐pass filtered between 20–500 Hz. EMG amplitude was rectified and averaged for the following phases during jumping (mEMG): before landing (100 ms preceding ground contact; Hoffren et al., 2007), in eccentric and concentric phases according to the ankle joint angle. In addition, the mean mEMG of MG, LG, and SOL was calculated as the mEMG of the plantar flexor muscles (PF). In the present study, we failed to obtain EMG data for one subject.

### 2.5 Statistical analysis

Descriptive data included means ± SD. The Kolmogorov–Smirnov test was used to verify the normal distribution of the measured variables. Joint stiffness at a 20‐cm drop height, jumping height at a 30‐cm drop height, tendon stiffness measured during ballistic contractions, and the ratios of mEMG before landing and during the eccentric phases to that during the concentric phase at all drop heights did not show a normal distribution, whereas the other measured variables showed a normal distribution. One‐way analysis of variance (ANOVA) with repeated measures was used to detect the significant effect of the drop heights (10, 20, and 30 cm) on the measured variables during the drop jump. If the F statistic of the analysis of variance was significant, differences among means were assessed using multiple comparisons with Bonferroni's correction. In ANOVA, Mauchly's sphericity test was performed to assess the homogeneity of variance. Greenhouse–Geisser correction was applied where the assumption of sphericity was violated. The effect size was calculated using partial eta‐squared (pη^2^) for one‐way ANOVA. To assess the relationships among measured parameters, Pearson's or Spearman's correlation coefficient according to data distribution was computed. The level of significance was set at *p* < 0.05. Statistical computations were performed using IBM SPSS Statistics (version 27).

## 3 RESULTS

Table 1 shows the measured variables during drop jumps at the three different drop heights. Peak torque (*p* < 0.001, pη^2^ = 0.802) and range of motion (*p* < 0.001, pη^2^ = 0.821) during the contact phase were significantly greater at higher drop heights, whereas joint stiffness was significantly lower at higher drop heights (*p* < 0.001, pη^2^ = 0.320). The angular velocities during the eccentric phase were significantly greater at higher drop heights (*p* < 0.001, pη^2^ = 0.926). No differences in mEMG during the eccentric (*p* = 0.986, pη^2^ = 0.001) and concentric (*p* = 0.497, pη^2^ = 0.025) phases or the ratios of mEMG before landing (*p* = 0.100, pη^2^ = 0.088) and during the eccentric phase (*p* = 0.240, pη^2^ = 0.056) to that during the concentric phase were found among the three different drop height conditions, whereas mEMG before landing was significantly greater at higher drop heights (*p* = 0.001, pη^2^ = 0.237). Jumping height was significantly greater at higher drop heights (*p* < 0.001, pη^2^ = 0.691).

**TABLE 1:** Measured variables during the drop jump at three drop heights Mean (SD).

	10‐cm drop height	20‐cm drop height	30‐cm drop height
Peak torque during contact phase (Nm)	130.8 (31.6)	159.0 (37.1) ***	179.9 (44.5) *** ^###^
Range of motion during contact phase (deg)	24.2 (5.7)	31.3 (4.9) ***	38.4 (4.8) *** ^###^
Joint stiffness (Nm deg^−1^)	5.7 (1.5)	5.3 (1.5)	4.8 (1.4) *** ^#^
Angular velocity during eccentric phase (deg s^−1^)	126.4 (20.8)	186.0 (28.4) ***	233.7 (29.8) *** ^###^
mEMG before landing (mV s^−1^)	0.06 (0.03)	0.07 (0.03)	0.08 (0.03) **
mEMG during eccentric phase (mV s^−1^)	0.10 (0.04)	0.10 (0.03)	0.10 (0.04)
mEMG during concentric phase (mV s^−1^)	0.08 (0.04)	0.07 (0.03)	0.07 (0.03)
Ratio of mEMG before landing to that during concentric phase	1.05 (0.82)	1.21 (0.99)	1.20 (0.78)
Ratio of mEMG during eccentric phase to that during concentric phase	1.70 (1.29)	1.70 (1.21)	1.57 (1.03)
Jumping height (cm)	16.9 (4.1)	19.2 (4.6) ***	21.5 (4.7) *** ^###^

* Significantly different from 10‐cm drop height (**p < 0.01, ***p < 0.001).

^#^ Significantly different from 20‐cm drop height (^#^ *p* < 0.05, ^###^ *p* < 0.001).

**FIGURE 3:**
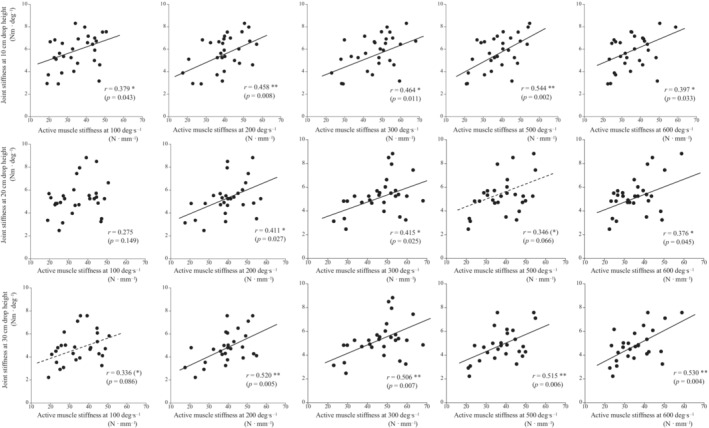
Relationships between joint stiffness at 10‐cm (upper panels), 20‐cm (middle panels), and 30‐cm (lower panels) drop heights and active muscle stiffness at five different angular velocities. (*) *p* < 0.10, *p < 0.05, **p < 0.01.

**FIGURE 4:**
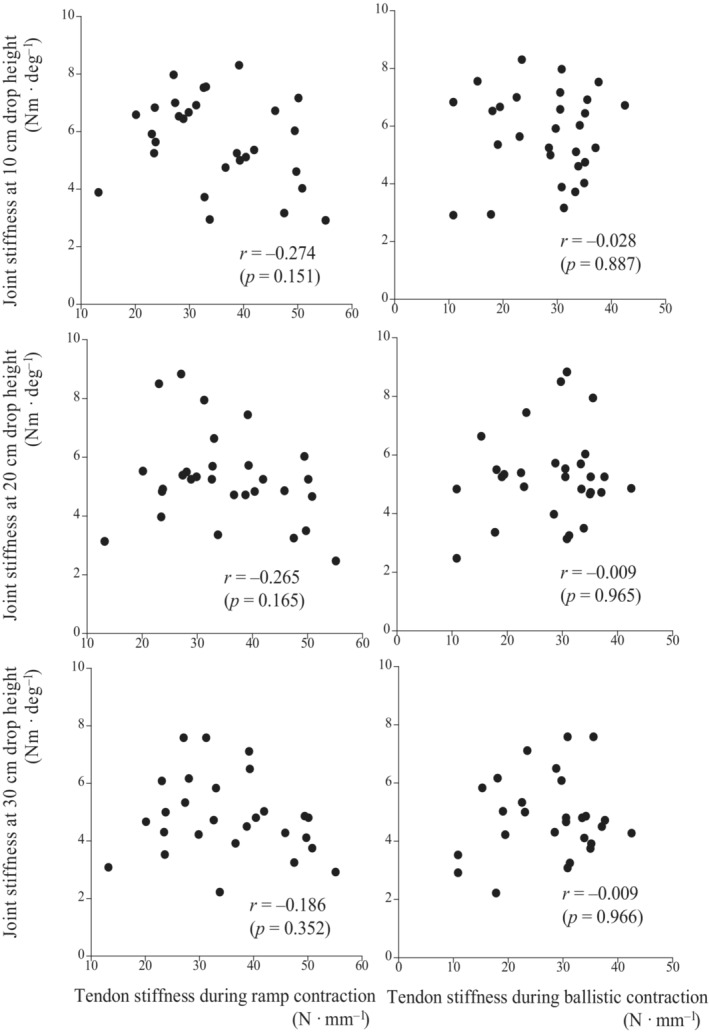
Relationships between joint stiffness at 10‐cm (upper panels), 20‐cm (middle panels), and 30‐cm (lower panels) drop heights and tendon stiffness measured during ramp (left panels) and ballistic (right panels) contractions.

**FIGURE 5:**
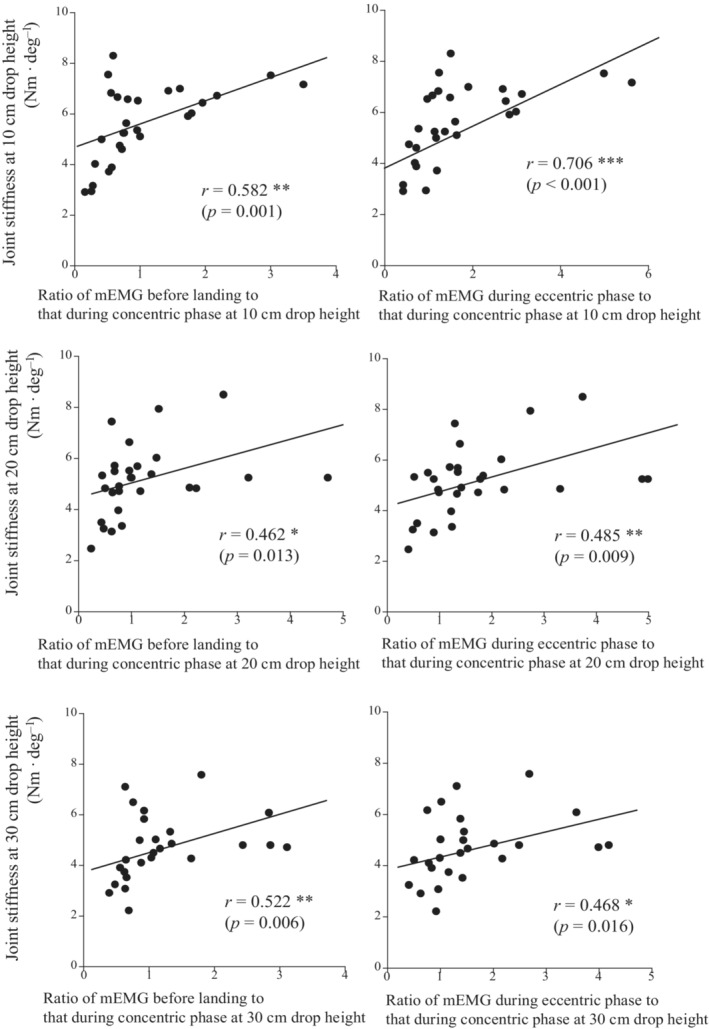
Relationships between joint stiffness at 10‐cm (upper panels), 20‐cm (middle panels), and 30‐cm (lower panels) drop heights and the ratios of the electromyographic activity of the plantar flexor muscles before landing (left panels) and during the eccentric phase (right panels) to that during the concentric phase. *p < 0.05, **p < 0.01, ***p < 0.001.

Active muscle stiffness at five different angular velocities was significantly correlated with joint stiffness at the three different drop heights, except for the relationships between joint stiffness at a 20‐cm drop height and active muscle stiffness at 100 and 500 deg s^−1^, and between joint stiffness at a 30‐cm drop height and active muscle stiffness at 100 deg s^−1^ (Figure 3). Tendon stiffness measured during ramp and ballistic contractions was not significantly correlated with joint stiffness at three different drop heights (Figure 4). The ratios of mEMG of the plantar flexor muscles before landing and during the eccentric phases to that during the concentric phase were significantly correlated with joint stiffness at the three different drop heights (Figure 5).

**FIGURE 6:**
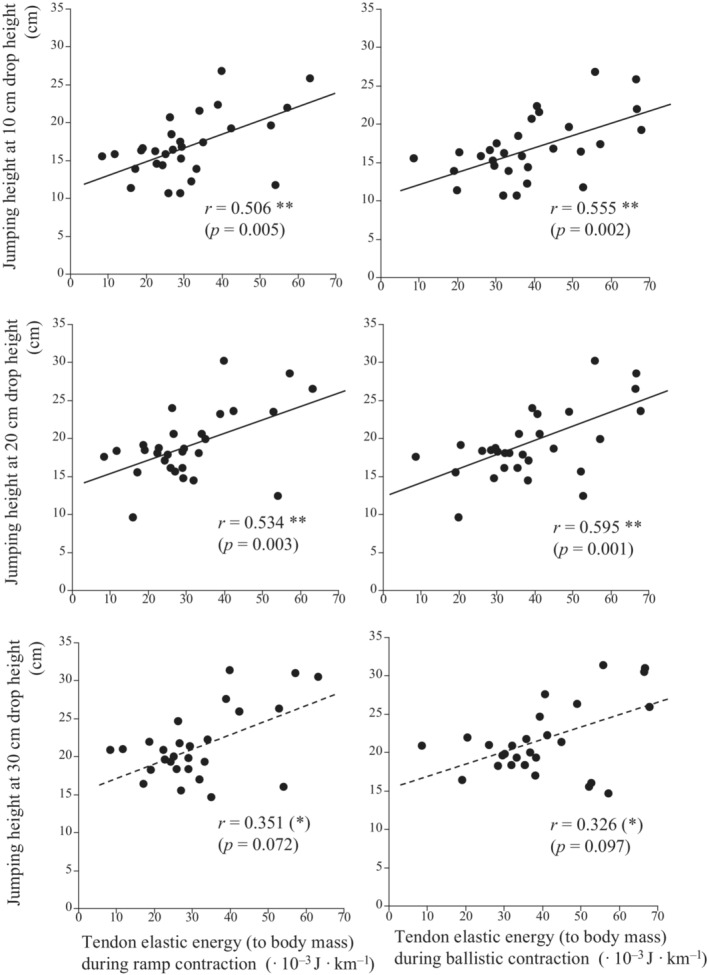
Relationships between jumping heights at 10‐cm (upper panels), 20‐cm (middle panels), and 30‐cm (lower panels) drop heights and tendon elastic energy measured during ramp (left panels) and ballistic (right panels) contractions. (*) *p* < 0.10, **p < 0.01.

Jumping heights at 10‐ and 20‐cm drop heights (except for 30 cm) were highly correlated with tendon elastic energy measured during ramp and ballistic contractions (Figure 6). No other measured variables showed significant correlations with jumping heights at any of the drop heights (Table 2).

**TABLE 2:** Correlation coefficients between jumping height and the measured variables.

	10‐cm drop height	20‐cm drop height	30‐cm drop height
Joint stiffness	−0.055	0.024	0.216
Active muscle stiffness at 100 deg s^−1^	0.026	0.099	0.059
Active muscle stiffness at 200 deg s^−1^	−0.060	0.012	−0.137
Active muscle stiffness at 300 deg s^−1^	0.078	0.154	0.087
Active muscle stiffness at 500 deg s^−1^	−0.071	−0.002	−0.013
Active muscle stiffness at 600 deg s^−1^	0.184	0.172	0.233
Maximal Fm (to body mass) during ramp contraction	0.324 (*)	0.364 (*)	0.097
Tendon stiffness during ramp contraction	0.008	0.065	−0.09
Tendon elastic energy (to body mass) during ramp contraction	0.506 **	0.534 **	0.351 (*)
Maximal Fm (to body mass) during ballistic contraction	0.341 (*)	0.378 *	0.045
Tendon stiffness during ballistic contraction	−0.050	−0.057	−0.030
Tendon elastic energy (to body mass) during ballistic contraction	0.555 **	0.595 **	0.326 (*)
Ratio of mEMG before landing to that during concentric phase	−0.275	−0.184	−0.063
Ratio of mEMG during eccentric phase to that during concentric phase	−0.100	−0.024	0.218

(*) *p* < 0.10, *p < 0.05, **p < 0.01.

## 4 DISCUSSION

The main results of the present study were that (1) joint stiffness was associated with active muscle stiffness and electromyographic activity patterns, but not tendon stiffness, and (2) jumping height (except for 30‐cm drop height) was closely correlated with tendon elastic energy, but not the other measured variables of muscle‐tendon mechanical properties or electromyographic activity.

Joint stiffness was significantly correlated with active muscle stiffness, although the correlation coefficients with muscle stiffness at 100 deg s^−1^ tended to be slightly lower compared with those at other angular velocities (Figure 3). Our previous studies showed that joint stiffness and active muscle stiffness changed similarly with transient fatigue tasks and long‐term training (Kubo et al., 2017, 2021; Kubo & Ikebukuro, 2019). Based on these results, we expected high correlations between joint stiffness and active muscle stiffness, and the present results support this hypothesis. The low correlation coefficients between joint stiffness and active muscle stiffness at 100 deg s^−1^ would be related to the difference in angular velocities between the measurements of active muscle stiffness and drop jump. At the beginning of the present study, we expected to identify high correlations between joint stiffness and active muscle stiffness under conditions similar to the angular velocity during the eccentric phase of drop jump, but unfortunately, that hypothesis was rejected. However, the correlation coefficients between joint stiffness at the 30‐cm drop height and muscle stiffness at higher angular velocities (300, 500, and 600 deg s^−1^) tended to be higher than those at the 10‐ and 20‐cm drop heights, which may partially support our hypothesis.

The relationship between tendon stiffness measured during ramp contractions and joint stiffness was consistent with the results of our previous study (Kubo et al., 2007). In the present study, tendon stiffness measured during ballistic contractions, in which the tendon strain rate was similar to that during the actual exercises, was also unrelated to joint stiffness (Figure 4). Our previous studies demonstrated that tendon stiffness measured during ramp and ballistic contractions did not change after repeated hopping exercises or 12 weeks of plyometric training, although joint stiffness and active muscle stiffness changed with both interventions (Kubo et al., 2017, 2021; Kubo & Ikebukuro, 2019). Considering these previous and present results, we concluded that tendon stiffness was unrelated to joint stiffness.

Previous studies demonstrated that joint stiffness was associated with electromyographic activity before landing and during the eccentric phase of drop jump (Hoffren et al., 2007; Horita et al., 2002). In the present study, the ratios of mEMG before landing and during the eccentric phase to that during the concentric phase were significantly correlated with joint stiffness at all drop heights (Figure 5). Therefore, the results of this study support the findings of previous studies. Marked electromyographic activity in the plantar flexor muscles before landing and during the eccentric phase increased joint stiffness and also stored more elastic energy in the Achilles tendon. Since the correlation coefficients between the electromyographic activity patterns and joint stiffness were highest for drop jumps with a drop height of 10 cm (Figure 5), electromyographic activity may be strongly related to joint stiffness during drop jump with a drop height of 10 cm. On the other hand, a trend toward higher correlation coefficients with active muscle stiffness at a high angular velocity has been observed for drop jump with a drop height of 30 cm (Figure 3). As the drop height increases, the contribution of electromyographic activity to joint stiffness may decrease, and the contribution of active muscle stiffness may increase.

Several studies showed that joint stiffness was related to running speed (Arampatzis et al., 1999; Kuitunen et al., 2002). However, surprisingly few studies have examined the relationship between joint stiffness and jumping height. We previously reported that jumping height was unrelated to joint stiffness (Kubo et al., 2007), and the same results were obtained in this study. In our previous study, we considered that joint stiffness might be strongly associated with performance during repetitive stretch‐shortening cycle exercise (e.g., running and hopping) rather than with that during a single movement (e.g., vertical jumping). In future studies, we need to examine the differences in the relationship between joint stiffness and performance with various modes of exercise.

Another interesting finding of this study was that the jumping height was closely correlated with tendon elastic energy, except for the drop height of 30 cm. Over the last two decades, the effects of tendon mechanical properties on performance and efficiency during stretch‐shortening cycle exercises have been investigated (Arampatzis et al., 2007; Bojsen‐Moller et al., 2005; Kubo et al., 2007). In these studies, tendon stiffness was generally used as the primary indicator of tendon mechanical properties. However, as noted in Introduction, jumping height (corresponding to the integral of the reaction force in the push‐off phase during jumping) is considered to be strongly associated with the amount of energy exerted by the muscle‐tendon complex. The results for drop jumps in this study also support this idea.

In the present study, there were some limitations with the methodology. Firstly, only three drop heights could be set in the present study. Several studies on drop jumps examined performance at more drop heights and provided optimal drop heights (Hakkinen et al., 1986; Komi & Bosco, 1978). In this study, we planned to follow these previous studies and perform measurements under several drop height conditions in order to verify the relationship between the optimum drop height and measured variables (muscle‐tendon mechanical properties and electromyographic activity). However, most subjects were unable to perform the task at drop heights greater than 30 cm (in fact, even at the 30‐cm drop height, two of the 29 subjects were unable to perform the task). Secondly, trials in which the knee joint did not remain at full extension during the drop jump were included. In the present study, trials in which the knee joint was flexed at more than 10 deg were judged to be failures, and additional measurements were taken. Thus, all trials involved less than 10‐deg knee joint flexion. Thirdly, all participants were men in the present study. Our results for men may differ from those for women since gender differences are known to exist regarding the mechanical properties of muscles and tendons (Kubo et al., 2003; Morse, 2011; Onambele et al., 2007).

In conclusion, the present results indicate that joint stiffness is determined by active muscle stiffness and electromyographic activity patterns during jumping, while jumping height is determined by tendon elastic energy. Furthermore, joint stiffness was not related to jumping height, which is generally accepted as a performance measure of stretch‐shortening cycle exercise.

## AUTHOR CONTRIBUTIONS

Takehiro Kosaka and Keitaro Kubo conceived and designed research; Takehiro Kosaka, Shuhei Sasajima, and Soushi Mino performed experiments; Takehiro Kosaka and Ayaka Yasuda analyzed data; Takehiro Kosaka, Soushi Mino, and Keitaro Kubo interpreted the results of experiments; Takehiro Kosaka, Shuhei Sasajima, and Ayaka Yasuda prepared tables and figures; Takehiro Kosaka and Keitaro Kubo drafted the manuscript; Takehiro Kosaka, Shuhei Sasajima, Ayaka Yasuda, Soushi Mino, and Keitaro Kubo edited and revised the manuscript; Takehiro Kosaka, Shuhei Sasajima, Ayaka Yasuda, Soushi Mino, and Keitaro Kubo approved the final version of manuscript.

## CONFLICT OF INTEREST STATEMENT

We have no conflict of interest with this work.

## ETHICS STATEMENT

This study was approved by the Ethics Committee for Human Experiments, Department of Life Science (Sports Sciences), The University of Tokyo.
